# Use, understanding, and perceptions of gut microbiome research and practices in oncology

**DOI:** 10.1007/s00520-026-11042-w

**Published:** 2026-08-12

**Authors:** Courtney B. Cross, Maya R. Davies, Elin Irestorm, Clifton P. Thornton, Caitlin W. Elgarten, Hannah R. Wardill, Madison T. Stein, Yin Ting Cheung, Dori M. Beeler, Jason L. Freedman

**Affiliations:** 1 School of Pharmacy and Biomedical Sciences, College of Health, Adelaide University, Adelaide, Australia; 2 Precision Medicine, South Australian Health and Medical Research Institute, Adelaide, Australia; 3 Department of Paediatrics, Faculty of Medicine, Lund University, Lund, Sweden; 4 Behavioural Sciences Unit, Discipline of Paediatrics & Child Health, School of Clinical Medicine, Randwick Clinical Campus, UNSW Medicine & Health, UNSW, Sydney, Australia; 5 Center for Pediatric Nursing Research & Evidence-Based Practice, Children’s Hospital of Philadelphia, Philadelphia, USA; 6 Division of Oncology, Department of Pediatrics, Children’s Hospital of Philadelphia, University of Pennsylvania School of Medicine, Philadelphia, USA; 7 Rowan-Virtua School of Osteopathic Medicine, Stratford, New Jersey USA; 8 School of Pharmacy, Faculty of Medicine, The Chinese University of Hong Kong, Hong Kong SAR, China; 9 Hong Kong Hub of Obstetric and Paediatric Excellence, The Chinese University of Hong Kong, Hong Kong SAR, China; 10 Department of Supportive Oncology, Atrium Health Levine Cancer, Charlotte, USA

**Keywords:** Gut microbiome, Medical oncology, Pediatric oncology, Supportive oncology

## Abstract

**Purpose:**

The gut microbiome has gained increasing recognition as an important factor influencing cancer treatment efficacy and toxicity. However, translation of this knowledge to deliver clinical benefit has been hampered by inconsistent approaches to both biospecimen sampling and investigation of microbial-based interventions, potentially reflecting inherent biases and/or barriers across research environments. Accordingly, we aimed to characterize the current practices and perceptions surrounding the gut microbiome among members of the Multinational Association of Supportive Care in Cancer (MASCC).

**Methods:**

A mixed-methods survey, probing current research and/or clinical use, plans, and perceptions of the gut microbiome in oncology, was developed in 2022 and distributed MASCC-wide via REDCap. Quantitative data were summarized using descriptive analyses, and qualitative responses were independently dual-coded using inductive thematic analysis.

**Results:**

Out of 117 respondents, the majority (83%) held the belief that the gut microbiome plays an important role in disease states such as cancer. 44.4% of survey respondents reported ongoing research or clinical practice regarding the gut microbiome at their center, with 70.5% also expressing an interest in developing such a program. Qualitative analysis revealed 6 main themes: (1) state of the evidence, (2) significance of the microbiome, (3) uncertainty, (4) aspirations, (5) preferences, and (6) critique.

**Conclusions:**

This survey identifies general concordance on the importance of the gut microbiome in cancer. Despite interest in this field, there is a clear need for resource support (i.e., funding and infrastructure) and increased expertise to support high-quality trials generating the evidence required for implementation of microbial-based sampling or therapeutics in clinical practice.

**Supplementary Information:**

The online version contains supplementary material available at https://doi.org/10.1007/s00520-026-11042-w.

## Introduction

The human gastrointestinal tract is home to a rich ecosystem of microorganisms, including bacteria, fungi, and viruses. These microbes, both in isolation - termed the gut *microbiota*, and together with their collective genomes, metabolites, and environment - termed the gut *microbiome* [1], are highly individualized, with their composition reflecting the cumulative impact of numerous host, environmental, and lifestyle factors such as diet, geography, disease, and medication. This unique attribute of the microbiome, whereby its composition acts as a conduit between a person’s nature, nurture, and their development of disease, coupled with the ease with which it can be manipulated, has seen it gain significant traction as a predictive and modifiable factor in disease, especially within the era of personalized medicine [2, 3].

A growing body of literature suggests that the gut microbiome influences cancer across the entire disease continuum, and, central to this role, is the immense control the microbiome exerts over the host’s immune system [4]. And so, while the reported mechanisms by which the gut microbiome influences cancer development, treatment response, survivorship, and relapse are diverse, a central theme across studies is its capacity to shape immune tone, balancing pro- and anti-inflammatory signaling as well as immune tolerance and anti-tumor immunity [5].

While the distinct microbial features displayed by people with cancer are increasingly reported, the causality of the gut microbiome in cancer development remains uncertain. In contrast, the microbiome’s role in shaping treatment response, including efficacy and toxicity, is described with increasing precision [6]. Across conventional and emerging modalities, cancer treatments disrupt the gut microbiome, depleting commensal microbes, reducing diversity, and permitting pathogen expansion. These compositional shifts *after treatment*, as well as microbiome composition *before treatment*, have been shown to contribute to therapeutic efficacy [7, 8]. For example, a microbiome-dependent response to immunotherapy has been demonstrated both preclinically and in patient cohorts [9–11].

The gut microbiome has also been implicated in numerous cancer treatment side effects, spanning multiple organ systems. Most notably, gastrointestinal (GI) complications such as oral and GI mucositis, diarrhea, infection, and graft-versus-host disease have clear microbial involvement [12, 13]. More recently, the gut microbiome’s role in neuropsychological side effects, such as cognitive impairment, has gained traction, underscoring its influence beyond the gut [14, 15]. In both contexts, studies have explored the microbiome’s potential as a predictive tool to explain variability in toxicity and as a therapeutic target to reduce symptom burden.

While the microbiome’s role across the cancer disease continuum is described with increasing precision within the literature, clinical translation of this knowledge has been limited. Successful translation into routine oncology care will depend not only on the strength of scientific evidence but also on the knowledge, perceptions, and readiness of clinicians to integrate emerging findings into practice. Healthcare professionals play a critical role in determining whether microbiome-related interventions are discussed with patients, incorporated into clinical decision-making, and prioritized for future research. Additionally, clinical knowledge, perceptions, and practices are likely to be highly heterogenous based on factors such as patient age (e.g., pediatric versus adult oncology) and geographic location. To date, the international landscape of microbiome-related knowledge and practices in oncology has not been investigated, a feat required to identify barriers to implementation, highlight areas requiring education or consensus, and inform strategies to facilitate the responsible translation of microbiome science into clinical oncology. Thus, we aimed to characterize the current knowledge, practices, and perceptions surrounding the gut microbiome in clinicians and researchers globally.

## Methods

### Survey methodology

A working group within the Multinational Association of Supportive Care in Cancer (MASCC) developed a mixed-methods survey (see Supplementary Material) integrating both quantitative and qualitative data to characterize global understanding, perceptions, and practices relating to the gut microbiome in oncology research as well as pediatric and adult clinical care. MASCC membership includes representation of healthcare professionals (i.e., oncologists, nurses, allied health), researchers, educators, industry representatives, and patient partners who focus on supportive care. The survey was distributed MASCC-wide in early 2022 to all current members (approximately 2600 recipients) and remained open for 2 months, with reminders sent at 2 weekly intervals. Surveys were distributed via email, and a single response per respondent was collected via REDCap. The survey captured the respondents’ demographic details, practice setting, and the research undertaken and/or clinical care patients receive at their institution, as well as their individual understanding and perceptions. All survey items were optional, and thus, respondents were not required to answer every question. Quantitative analyses were performed using available responses for each survey item, and percentages were calculated using the corresponding item-specific denominator with no imputation undertaken. Qualitative analyses included all free-text responses submitted for each open-ended question, with only the responses provided to that question included in the thematic analysis. This study was approved by the Institutional Review Board at the Children’s Hospital of Philadelphia and was deemed exempt from full review (IRB 22–019620).

### Data analysis

This study employed a convergent parallel design, where quantitative and qualitative data were collected simultaneously, analyzed separately, then merged to capture both the breadth and depth of respondents’ perspectives across diverse global contexts.

#### Quantitative analysis

All quantitative data were collected using descriptive parameters (e.g., demographics) or multiple-choice style questions (e.g., yes/no). Descriptive analyses were conducted using GraphPad Prism (version 10), with percentages calculated to summarize binary and categorical data.

#### Qualitative analysis

Qualitative responses to open-ended survey items were independently, dual-coded by CC, MD, and EI using team-based iterative-inductive thematic analysis in NVivo (version 15) [16, 17], creating harmony among multiple coders through focusing on consensus. Team members met to compare the different theme trees, identifying similar and distinct codes which could be grouped or remain separate, respectively. When coding discrepancies were identified, intercoder consensus was reached through discussion among all team members. Preliminary codebooks were drafted based on theme trees and revised until agreement was reached that no new themes were emerging and no difficulties were encountered with existing themes. Codes were deliberated, refined, and used to generate main themes. These were organized into the highest level, with themes combined across the questions and further stratified by yes or no answers provided. Respondents are represented according to their study ID.

## Results

### Respondent demographics

A total of 117 responses were obtained from approximately 2600 international recipients yielding an overall response rate of 4.5%. Of these, 48 responses were complete and 69 were partially complete with at least one response to one survey item omitted. As survey items were optional, the number of responses varied across items with both complete and partial responses included in analyses. Therefore, the denominator for each analysis reflects the number of respondents who answered the respective survey item. Respondents were from five continents, representing a range of occupations across clinical and research oncology, including physicians, academic researchers, dentists, allied health professionals, nurse practitioners, and pharmacists. Respondents were based primarily in a hospital setting with specialties spanning adult (*N* = 40) and pediatric (*N* = 11) oncology/stem cell transplantation (Supplementary Table 1). The respondents’ primary centers saw an average of 2900 new patients each year (minimum of 20; maximum of 40,000) and performed 170 stem cell transplants (minimum of 0; maximum of 5000). Of these centers, 76% had an antimicrobial stewardship program and 55% had a specific immunocompromised infectious disease service.

### Quantitative results

#### Current practice relating to the gut microbiome in clinical oncology and research

**Fig. 1:**
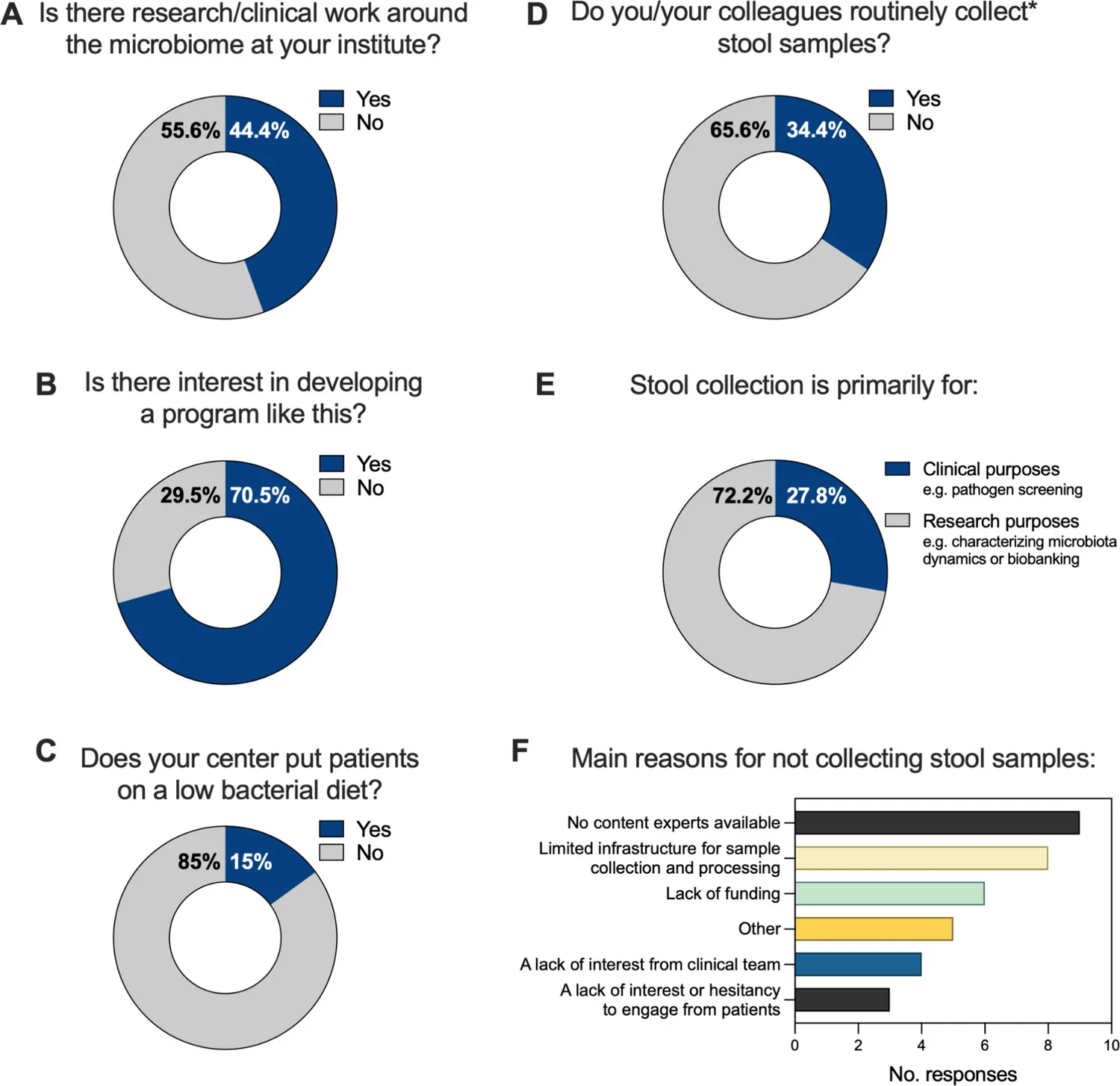
Current practice in gut microbiome research. **A** 63 responses; **B** 34 responses; **C** 60 responses; **D** *for gut microbiome surveillance or research, 61 responses; **E** 18 responses, **F** 35 responses. Percentages were calculated using the number of respondents who answered each survey item as the denominator. This approach ensured that both complete and partially completed questionnaires were included in the analyses, while accounting for the optional nature of the survey items

44.4% of survey respondents reported ongoing research or clinical practice regarding the gut microbiome at their center (Fig. 1A); however, 70.5% also expressed interest in developing such a program (Fig. 1B). When expanding on motivations or hesitancy to engage with microbiome research, modifiable issues such as lack of funding and expertise were expressed as key barriers to developing such programs. This contrasted with less easily modified barriers (i.e., lack of interest from clinicians and patient perceptions), which received fewer responses. Regarding clinical practice, 15% of respondents reported the ongoing use of a low-bacterial diet at their center (Fig. 1C).

One-third of respondents (34.4%) routinely collected stool from patients (Fig. 1D), with the leading purpose in this subset being research (72.3%, e.g., characterizing microbiota dynamics or biobanking) (Fig. 1E). In line with the barriers to engaging with microbiome research identified above, a lack of content experts and limited infrastructure were also highlighted as the top two barriers impeding routine stool collection, with hesitancy and/or lack of interest from the clinical team and patients receiving fewer responses (Fig. 1F).

**Fig. 2:**
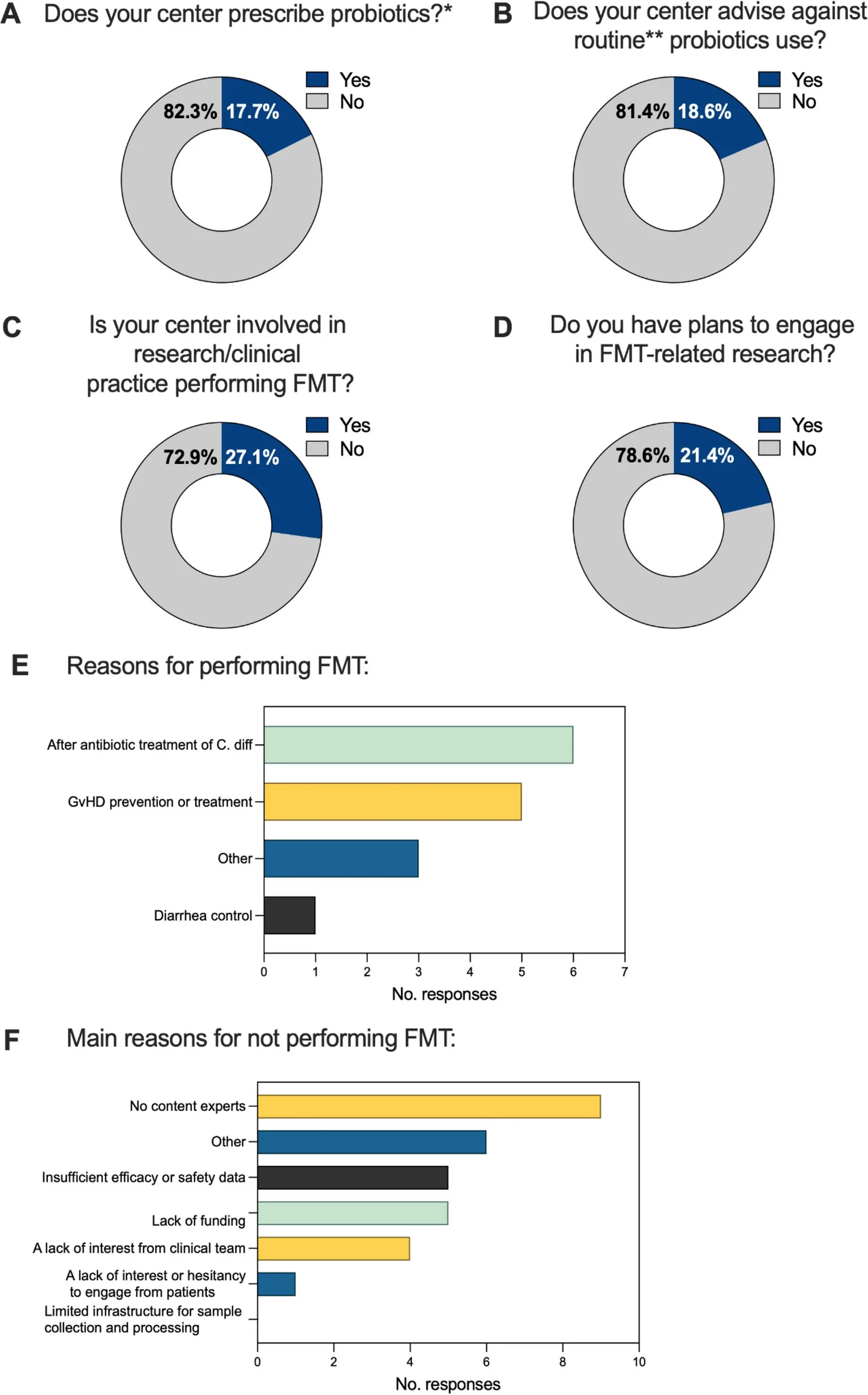
Current use of interventions targeting the gut microbiome. **A** *For standard of care or as part of a clinical trial, 62 responses; **B** **Aside from yoghurt, 62 responses; **C** 59 responses; **D** 42 responses, **E** 15 responses, **F** 25 responses. Percentages were calculated using the number of respondents who answered each survey item as the denominator. This approach ensured that both complete and partially completed questionnaires were included in the analyses, while accounting for the optional nature of the survey items

Uptake of gut microbiota-targeted interventions was also surveyed. A similar number of respondents reported currently prescribing probiotics as those who advised against their use (17.7% and 18.6%, respectively, Fig. 1A and B). Less than one-third (27.1%) of centers were currently performing fecal microbiota transplantation (FMT) either for research or clinical purposes (Fig. 2C), while 21.4% of respondents reported having plans to engage in FMT-related research (Fig. 2D). The leading indication for FMT was antibiotic-refractory *Clostridium difficile* (*C. diff*) infection, aligning with multiple international guidelines for *C. diff* management. The prevention/treatment of graft versus host disease (GvHD), a potentially life-threatening complication of allogenic stem cell transplant, was the second most common FMT indication (Fig. 2E). Conversely, the main reason for not performing FMT was again “no content experts,” followed by “other” (Fig. 2F). Responses for “other” included reasons such as FMT is *not yet standard of practice* and that centers *are not set up for this type of work*, all of which are modifiable with greater support and infrastructure.

#### Current perceptions regarding the gut microbiota in supportive oncology

**Fig. 3:**
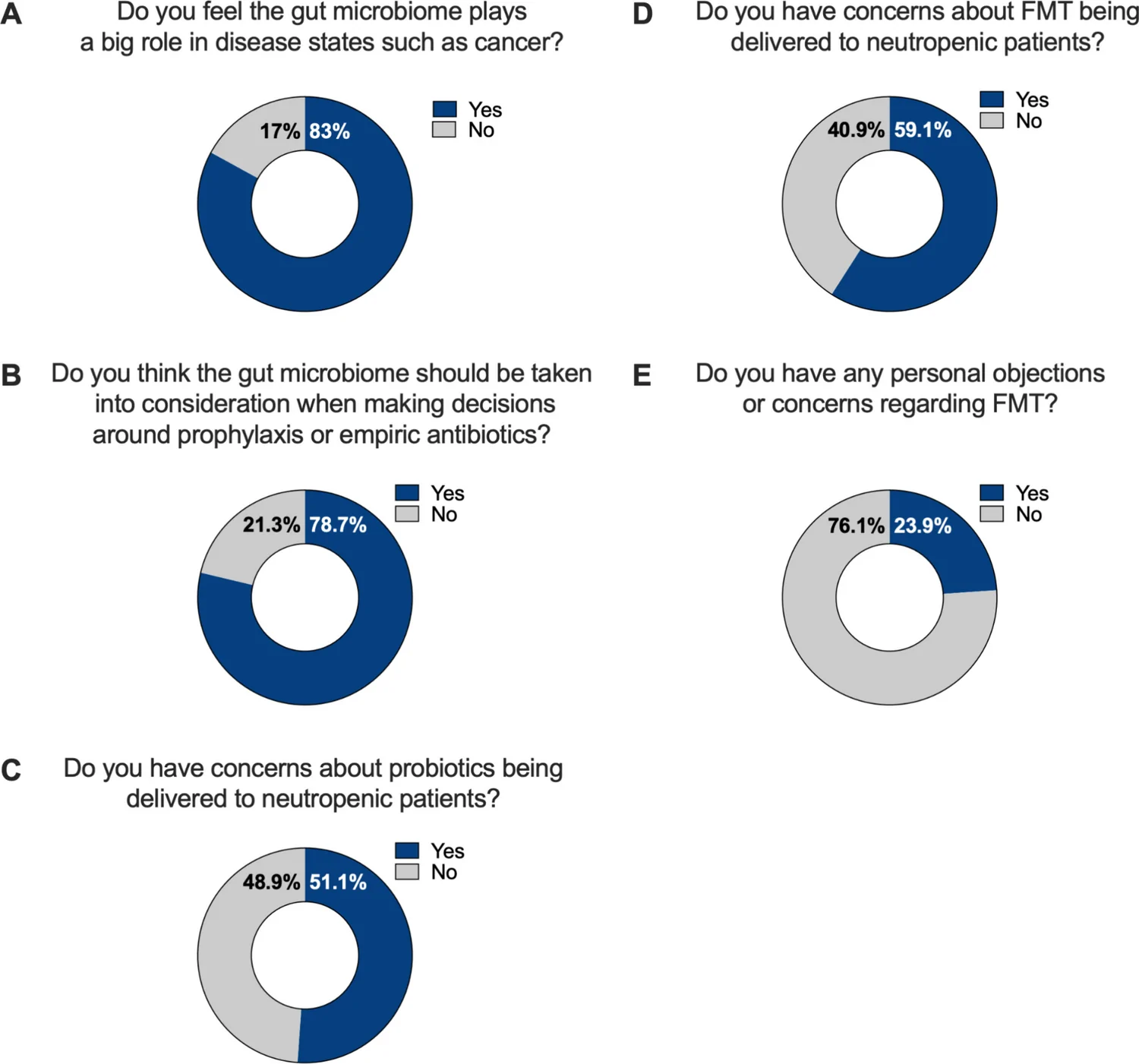
Perceptions around the gut microbiome in cancer. **A** 47 responses, **B** 47 responses, **C** 45 responses, **D** 44 responses, **E** 46 responses. Percentages were calculated using the number of respondents who answered each survey item as the denominator. This approach ensured that both complete and partially completed questionnaires were included in the analyses, while accounting for the optional nature of the survey items

The vast majority (83%) of survey respondents viewed that the gut microbiome plays an important role in disease states such as cancer (Fig. 3A). Similarly, most respondents thought it important to consider the impact on the gut microbiome when deciding whether to prescribe antibiotics for empiric or prophylactic purposes (Fig. 3B). Approximately half of the respondents (51.1%) identified concerns about probiotics being delivered to neutropenic patients (Fig. 3C), and a larger proportion (59.1%) expressed concerns about FMT delivery to neutropenic patients (Fig. 3D). This contrasted with only 23.9% of respondents disclosing that they held personal objections for the use of FMT (Fig. 3E).

### Qualitative results

Across all qualitative survey items, four questions were identified as receiving an adequate number of responses for analysis. These included the following: (i) “Do you feel the gut microbiome plays a big role in disease states such as cancer”; (ii) “Do you think the microbiome should be taken into consideration when making decisions around prophylaxis or empiric antibiotics”; (iii) “Do you have any personal objections or concerns regarding fecal microbiota transplantation (FMT)”; and (iv) “Do you have concerns about probiotics being delivered to neutropenic patients.”

Across these questions, the codes generated resulted in six main themes: (1) *State of the evidence*, (2) *Significance of the microbiome*, (3) *Uncertainty*, (4) *Aspirations*, (5) *Preferences*, and (6) C*ritique* (Table 1). The theme “significance of the microbiome” was further grouped into four subthemes: (a) *Significant-cancer*, (b) *Significant-immune response*, (c) *Significant-both*, and (d) *Significant-other*. Survey questions were preceded by a quantitative, multiple-choice style question (e.g., yes/no). Therefore, the following themes contain ideas under both yes and/or no perspectives. Selected representative quotations and the full set of quotations under each theme and subtheme are presented in Table 2 and Supplementary Table 2, respectively.

**Table 1:** Qualitative data themes, subthemes, and their definitions

Theme	Subtheme	Respondents reporting				Definition
		Q11	Q12	Q13	Q15	
State of the evidence		8	18	8	This theme reflects qualitative responses that rely on evidence to support their statements. This evidence is derived from research literature, practice experience, or other forms of professional expertise	18
Significance of the microbiome		20	0	0	This parent theme represents responses detailing the significant role of the microbiome across several subthemes, including Significant-Cancer, Significant-Immune Response, Significant-Both, and Significant-Other Disease	0
	Significant-Cancer	4	0	0	Responses detailing the significant role that the microbiome serves in cancer biology and/or care	0
	Significant-Immune Response	9	0	0	Responses detailing the significance of the microbiome in an immunomodulatory role	0
	Significant-Both	5	0	0	Responses detailing the significance of the microbiome in the capacity of an immunomodulatory role and in cancer biology and/or care	0
	Significant-Other Disease	2	0	0	Responses detailing the significant role that the microbiome serves in other disease biology and/or care	0
Uncertainty		5	3	3	Uncertainty reflects responses that convey a sense of doubt or ambiguity on something due to a lack of knowledge	0
Aspirations		2	2	6	Responses that reflect an interest in or ambition for working with the microbiome in practice, in research, or in grant work	3
Preferences		0	2	2	Responses that reflect preferences for how the microbiome should be integrated into daily practice, research, etc	3
Critique		0	2	6	General critiques expressing concerns over the microbiome in practice or authoritative ideas about how the microbiome should be used in practice	2

*Q11* Do you feel the gut microbiome plays a big role in disease states such as cancer, please explainyour thoughts; *Q12* Do you think the microbiome should be taken into consideration when makingdecisions around prophylaxis or empiric antibiotics, please explain your thoughts; *Q13* Do you haveany personal objections or concerns regarding fecal microbiota transplantation (FMT), please explainyour perspectives; and *Q15* Do you have concerns about probiotics being delivered to neutropenicpatients, what has informed this perspective

**Table 2:** Representative quotes used for analysis, organized by theme

Theme	Subtheme	Sample quotes
**State of the Evidence**		• Further clinical trials are required to identify the specific conditions required for each disease state, but this is a promising new technology for restoring microbiome following some sort of insult, i.e. antibiotics, disease, cancer treatment, etc., P27 (Q13) • Mounting evidence suggests importance, P14 (Q11) • I have had patients who insist on taking probiotics develop bacteremia from the same bug, P7 (Q15)
**Significance of the microbiome**	*Significant-Cancer*	• I think [it has a] huge impact on supportive care and late effects - inflammatory changes drive a lot of the complications (fatigue) and we don't understand [the] mechanism[s] …, P35 (Q11)
	*Significant-Immune Response*	• Gut microbiome interacts with mucosal innate immune mediators, leading to up or down regulation of immune and inflammatory responses with changes. There is now a lot of evidence to show [the] gut microbiome is involved in not only cancer, but respiratory diseases, neurological conditions (coming about through gut-brain axis), diabetes, obesity, and a whole range of other diseases. It also plays a big role in treatments for diseases, affecting metabolism of many drugs (i.e. is required for activation of some drugs), P26 (Q11)
	*Significant-Both*	• The microbiota is a key player in cancer pathogenesis, onset and progression. As regards breast cancer, its involvement includes the host's metabolism, immunity and hormonal balance through estrobolome P3 (Q11)
	*Significant-Other Disease*	• There is always lots of excitement around new fields, especially when the data are complex. I think the microbiome is important for what's happening in the GI tract, but I am skeptical that the relationship between the microbiome and systemic disease is going to be meaningful, P5 (Q11)
**Uncertainty**		• I really don’t know, P20 (Q11) • I don’t see this relationship clinically, P29 (Q11)
**Aspirations**		• Maybe worth a shot, P16 (Q12) • I am not against this practice, but personally, I should study more about it, P17 (Q15)
**Preferences**		• Depends on the threshold and setting, e.g. mildly neutropenic patient in outpatients would be fine with probiotics but I would not administer these in profoundly neutropenic patients in ICU, given the literature, P10 (Q15) • It depends on how neutropenic and what probiotics, P8 (Q15)
**Critiques**		• Neutropenic patients should not be given bacterial loads, P18 (Q15) • We give antibiotics without considering the wider impact - and without then providing ‘rescue’ or even advice on restoring microbiome, P35 (Q12)

*Q11* Do you feel the gut microbiome plays a big role in disease states such as cancer, please explain your thoughts; *Q12* Do you think the microbiome should be taken into consideration when making decisions around prophylaxis or empiric antibiotics, please explain your thoughts; *Q13* Do you have any personal objections or concerns regarding fecal microbiota transplantation (FMT), please explain your perspectives; and *Q15* Do you have concerns about probiotics being delivered to neutropenic patients, what has informed this perspective

#### State of the evidence

*State of the evidence* captures responses that rely on evidence derived from research literature, practical expertise, or professional experience. This theme was supported by 52 responses, providing a rich dataset with views on both sufficient and insufficient evidence in the field. P12 reflected that “numerous studies have shown antibiotics (via their detrimental effects on the microbiome) negatively impact treatment outcomes in cancer,” which was aligned with P19’s view that “preliminary evidence suggests possible reduced efficacy of … immunotherapy with reduced gut microbiome diversity.” In contrast, a strong emphasis by several respondents was placed on the need for more research, as represented by P31 who shared “I don’t think that enough is known to make proper decisions,” aligning with numerous additional examples: (i) P1, “much more research is needed”; (ii) P27, “further clinical trials are required”; and (iii) P5, “not enough evidence, to my knowledge.”

#### Significance of the microbiome

The parent theme, *Significance of the microbiome*, captures responses detailing the microbiome’s role in human physiology and disease across four subthemes. *Significant cancer* encompasses the microbiome’s role in cancer biology and/or care, with P17 describing its involvement, “not only in developing tumors, but also in modulating the oncological response, toxicity and other parameters related to [cancer] drugs.” Similarly, the subtheme *Significant-immune response* represents the microbiome’s key immunomodulatory role and thus its influence across many disease states. P8 described that “ultimately, many disease states are influence[d] by aberrant immune responses [and given] the microbiome is the most influential factor shaping immune function … it is highly likely that the microbiome indirectly impacts many disease states.” *Significant-both* captures responses that describe the microbiome’s intersecting role across both cancer and immune response, supported by statements such as “it is related to development of cancer but also with the immune capacity” from P23. Lastly, the subtheme *Significant-other* captures responses detailing the importance of the microbiome in other disease states, where P7 shared “I do feel strongly about its role in diseases like C.diff [infection]” and P5 who viewed that the “microbiome is important for what’s happening in the GI tract.”

#### Uncertainty

*Uncertainty* was also described by respondents, reflecting a sense of doubt or ambiguity on a question due to a lack of personal knowledge. For example, P18 and P5 reflected on the use of microbiome-targeted therapeutics, including probiotics and FMT, in the cancer setting, stating that “as a med[ical] oncologist, I don’t decide on these issues, it becomes a matter for gastroenterology” and “if they work we should use them, not my area of expertise,” respectively.

#### Aspirations

*Aspirations*, encompassing an interest or ambition to integrate the microbiome into clinical practice and research programs, were expressed by respondents. These were largely in relation to the use of microbiome-targeted interventions, where P22 shared that they would be “willing to [investigate these], if we get the opportunity,” and P35 described that these are “all worth exploring - but within the context of a fully resourced clinical trial.”

#### Preferences

Respondents shared their *Preferences* on how the microbiome could be integrated into research and clinical practice. These were in the context of whether the microbiome should be taken into account when making decisions on antibiotic use, where P18 indicated that “if a patient is myelo-suppressed and has sepsis, antimicrobial[s] are lifesaving.” Likewise, if considering the use of probiotics and/or FMT in neutropenic patients, P19 felt “[my] preference remains diet modification.”

#### Critique

Lastly, the theme *Critique* captures general concerns or authoritative ideas surrounding the way in which the microbiome should be incorporated into cancer care and research. Considering the use of FMT, P31 shared concerns regarding “safety in immunocompromised patients,” with P7 also sharing their concern was primarily due to “possible translocation of bacteria into the bloodstream.” Critiques regarding the validity of microbiota targeted therapeutics were also shared, with P8’s view that FMT “[is] not sufficient as [an] isolated matter” and, conversely, P12 indicating that “probiotics are limited in their diversity … I would advocate for microbial interventions to be based on more complex microbial mixtures e.g. FMT.” Another key message supporting this theme was the necessity to consider the microbiome when prescribing antibiotics. For example, P35 shared that “we give antibiotics without considering the wider impact,” aligning closely with P10’s view that “we over-prescribe antibiotics … we need to be careful with antibiotic stewardship and consider risk-benefit properly.”

## Discussion

This mixed-methods analysis of survey responses highlights that the gut microbiome is generally well recognized to play an important role in cancer. Many responses described knowledge of evidence demonstrating the importance of the gut microbiome, particularly of microbiota-immune interactions impacting many disease states, including cancer. However, in parallel, respondents also expressed uncertainty about the current evidence or the importance of the microbiome, while disclosing highly dubious perceptions regarding the impact of the microbiome in oncology. Porcari et al. recently published a framework for translating microbiome research (all diseases) into clinical practice, which underscored the importance of communication between microbiome researchers and clinicians to achieve clinical impact [18]. This aligns with our findings indicating disparity in perceptions of the gut microbiome’s role in cancer, with the theme *Uncertainty* highlighting gaps in the delivery of research knowledge to clinical providers.

Many responses highlighted concerns around the safety of interventions relating to the gut microbiome, particularly in neutropenic patients such as during hematopoietic stem cell transplant (HSCT). These concerns highlight a gap in research implementation, with a recent systematic review of FMT use in allogeneic HSCT reporting no serious adverse events in the majority of the literature and indicating broadly good tolerability [19]. In the setting of immunocompromised *C. diff*, the disease context in which FMT has been investigated most extensively, rates of serious adverse events are comparable to rates in immunocompetent populations [20]. With published evidence indicating the gut microbiome is an important predictive component of allogeneic HSCT outcomes, such as overall survival [21], poor translation of these findings to clinical providers prevents the delivery of therapeutic benefit. Importantly, a key barrier to implementing microbiome research to achieve clinical impact is uncertainty among clinicians [18]. We saw this idea emerging in several responses, where individuals expressed a need to learn more or acknowledged that decisions were beyond their expertise. In fact, our quantitative analysis identified a lack of content experts as the key barrier to both collecting stool and performing FMT. In contrast, under themes of both *Preferences* and *Critiques*, some respondents demonstrated confidence in their knowledge base by describing their perspectives on important considerations (*Preferences*) or best practice (*Critiques*). In both settings, these practices may reflect the fast-moving field of microbiome research and the difficulty for clinicians to update practice to incorporate new findings rapidly. This could explain why practice norms appear more reflective of past knowledge, thereby preventing innovation in harnessing the microbiome to improve outcomes.

While a gap in implementation emerged, a need for more research evidence was also described with the overarching theme, *State of the Evidence*. Irrespective of respondents’ perspectives on safety, efficacy, or the importance of gut microbiome research, there was a consistent call for more high-quality evidence. Notably, there was a large (70.5%) desire for a research/specialty program. When asked about barriers to concrete clinical actions, respondents endorsed modifiable issues regarding resource availability (lack of experts and infrastructure) over harder-to-modify barriers (lack of interest from clinicians or patients). This presents an opportunity, with interested parties eager to address the need for higher-quality evidence to overcome barriers to clinical implementation. However, to facilitate this, greater infrastructure and research support need to be made available and accessible. Unfortunately, on a global scale, research relating to improved survivorship receives the lowest amount of funding within cancer research (~ 4%) [22]. A potential solution to address this problem is to leverage global networks, such as MASCC, to perform multi-center clinical trials and biobanking initiatives to share research expertise. Multi-center studies also have the added benefit of producing evidence that is more representative, generalizable, and inclusive of more populations. In addition, shared knowledge through collaborative undertakings promotes the standardization of approaches to intervention and analysis, improving the reproducibility of results.

To our knowledge, this is the first international survey to comprehensively characterize gut microbiome-related knowledge, perceptions, and clinical practices among clinicians and researchers in the oncology field. Although prior surveys have explored healthcare professionals’ awareness of the gut microbiome in broader clinical and population settings, as well as clinician perceptions of other emerging areas of oncology (e.g., genomics), none has examined the translational relevance of gut microbiome science within oncology [23, 24]. Thus, these findings provide novel insights into current knowledge perspectives and perceptions relating to the gut microbiome in oncology. However, there are also some limitations that should be considered when interpreting these findings. Firstly, the relatively low response rate (approximately 4.5%), and the nature of survey items being optional, may have introduced non-response bias, whereby individuals with a greater interest in the gut microbiome may have been more likely to participate. Consequently, the findings should be interpreted with caution, as this may limit the generalizability of the results and overestimate awareness, knowledge, or enthusiasm for microbiome research and its application in oncology. The majority of respondents worked in the adult setting and therefore the views expressed may not be representative of the pediatric context. However, it is likely that concerns of safety and limited evidence may also be present in pediatric-specific care providers given that pediatric cancer populations have received far less attention than adults in gut microbiome research [25]. In addition, the inclusion of academics/researchers within the study (14.3%) may have skewed some of the results, as they may be more aware of emerging evidence with less appreciation of practical considerations of clinical care. The small sample size of this study did not permit subgroup analysis, and therefore, we were unable to determine trends in responses from clinicians alone or in the pediatric context. Finally, while several yes/no questions had associated “please explain” open-ended questions, many received too few responses to perform thematic analysis. Therefore, the identified themes were drawn from the four questions that received a sufficient number and depth of response. However, the response rate to these four questions may reflect that these are related to key controversies or essential considerations within the field and hence were more stimulating for participants. Overall practices and perceptions in clinical oncology are clearly highly heterogeneous, and further research is needed to assess these differences within specific subgroups, such as by age or geographic location.

Despite heterogeneity in current perceptions, there was a clear interest in an enhanced understanding of the microbiome in cancer, with a majority (> 70%) calling for further work and a desire for specialty research programs. Considering this alongside the robust and growing body of knowledge describing a key role of the microbiome in cancer, for institutions aiming to stay at the forefront of cancer treatment, we advocate for hiring microbiome experts, building clinician-researcher collaborative consortia, and investing in the infrastructure to support microbiome-related work. Given that exploiting the gut microbiome for clinical benefit holds promise for supportive oncology, particularly with the growth of personalized approaches in cancer care, such research efforts will be critical for widespread clinical implementation of microbiome-targeted strategies to improve the quality of supportive cancer care on an international scale.

## Conclusions

This survey highlights general agreement on the importance of the gut microbiome, particularly of microbiota-immune interactions, in cancer across the disease continuum. Respondents showed significant interest in engaging further with the field, but paralleled uncertainty, safety concerns, and dubious perceptions regarding the impact of research require high-quality evidence to disperse. Thus, more research is required in this area to progress international clinical knowledge, perceptions, and translation of microbiome-related practices or therapeutic strategies within the field of cancer care. Importantly, there are people willing and eager to perform it; thus, greater access to research funding, expertise, and infrastructure has the promise to deliver significant impact.

## Supplementary Information

Below is the link to the electronic supplementary material.

ESM1 (PDF 188 KB)

## Data Availability

Data will be made available upon reasonable request from the corresponding author.
