# Supplementary material for: Use, understanding, and perceptions of gut microbiome research and practices in oncology
Source: Support Care Cancer. 2026 Aug 12;34(9):843. doi: 10.1007/s00520-026-11042-w (PMC13469402; doi:10.1007/s00520-026-11042-w)
Supplement: Supplementary file 1 [file 520_2026_11042_MOESM1_ESM.pdf]

SUPPLEMENTARY MATERIAL

Supplementary Table 1: Demographics of survey respondents

Geographic Location	North America	16 (36.4%)
	Asia	13 (29.5%)
	Europe	10 (22.7%)
	South America	4 (9.1%)
	Australia	2 (4.5%)
	Responses *	44 (37%)
Primary Occupation	Physician	38 (60.3%)
	Academic/Researcher	9 (14.3%)
	Dentist/Oral Health Specialist	8 (12.7%)
	Allied Health Professional	2 (3.2%)
	Nurse	2 (3.2%)
	Pharmacist	2 (3.2%)
	Student	2 (3.2%)
	Responses *	63 (54%)
Work Location	Hospital	31 (49.2%)
	University Hospital	14 (22.2%)
	University or Research Institute	12 (19.0%)
	Other	2 (3.2%)
	Private Clinic	2 (3.2%)
	Responses *	63 (54%)
Clinical Specialty	Adult Oncology	38 (60.3%)
	Pediatric Oncology	9 (14.3%)
	Adult Stem Cell Transplantation	2 (3.2%)
	Pediatric Stem Cell Transplantation	2 (3.2%)
	All the above	5 (8.0%)
	N/A	7 (11.1%)
	Responses *	63 (54%)

* Number of responses obtained for descriptive parameter (N / 117)

Supplementary Table 2: All respondents' quotes used for analysis organized by theme

THEME	SUB THEME	SAMPLE QUOTES	
		**No**	**Yes**
**State of the Evidence**		• Insufficient data. (Q11) P6<br>• Haven't seen evidence. (Q11) P11<br>• No mature data yet. (Q12) P2<br>• Maybe but we need more research. (Q12) P4<br>• I don't think that enough is known to make proper decisions. (Q12) P31<br>• not enough evidence, to my knowledge. (Q12) P5<br>Preliminary studies shows benefit. (Q13) P15<br>There are previous studies. (Q13) P24<br>• I know of a center and a professional that does stool transplantation and has good results. (Q13) P29<br>• *Further clinical trials are required to identify the specific conditions required for each disease state, but this is a promising new technology for restoring microbiome following some sort of insult, ie antibiotics, disease, cancer treatment, etc. (Q13) P27<br>• *All worth exploring - but within the context of a fully resourced clinical trial. (Q13) P35<br>• There are no specific concerns. (Q15) P22<br>• Lack of knowledge. (Q15) P5<br>• Limited research that I am aware of. (Q15) P15<br>• *Lack of cases to imply significant risk. (Q15) P19<br>• …there is still reluctance because of a trial that had adverse effects in immunocompromised patients. But this should not stop all research on probiotics in these patients. (Q15) P21<br>• Research in other immunocompromised groups. (Q15) P35<br>• *Lack of cases to imply significant risk. (Q15) P19	• I think there is a role, although much more research is needed. (Q11) P1<br>• Yes, but much to learn yet. (Q11) P2<br>• Mounting evidence suggests importance. (Q11) P14<br>• There is wide evidence in [the] literature. (Q11) P24<br>• See literature. (Q11) P27<br>• Emerging evidence supports this theory. (Q11) P31<br>• …to protect colonization resistance and opportunistic superinfections. (Q12) P27<br>• Yes, baseline stool with gut microbes is a fingerprint signature of an individual patient. baseline stool examination and culture will help us prior knowing sensitivity of antibiotics. (Q12) P34<br>• I say yes with a caveat, because we have studied using fluoroquinolones vs. no antimicrobial prophylaxis three times, and each time it didn't seem to have too much of an effect on GVH rates, but rates of bacteremia increased. Over time we have been able to curb our use of empiric antibiotics, especially vancomycin in the febrile neutropenia setting, and our rates of VRE colonization has dramatically decreased. (Q12) P7<br>…it should, but there is also a need to research on the matter to define also safety in either approach. (Q12) P28<br>• yes ideally but need results available quickly. (Q12) P32<br>• I believe that the absorption of antibiotics is fully related to the gut microbiome metabolism. (Q12) P25<br>• It will support the gut and reduce adverse events. (Q12) P22<br>• Antibiotics need to be carefully selected and impacts on gut microbiota taken into consideration. If you reduce the diversity of the gut microbiota with antibiotics, many of the beneficial functions and metabolites of gut microbiota are reduced, resulting in other issues developing. (Q12) P26<br>• Antibiotics can alter the gut flora and so yes, studying the microbiome can make a difference. (Q12) P29<br>• Changing microbiome compromise therapeutic response. (Q12) P29<br>• Numerous studies have shown antibiotics (via their detrimental effects on the microbiome) negatively impact treatment outcomes in cancer, increasing the risk of acute and chronic complications (e.g. GvHD). As such, antibiotic stewardship is critical. (Q12) P12

			• Some studies show, for example, that different antibiotics can be associated with worse response to immunotherapy, and this could be related to microbiome. (Q12) P17<br>• Preliminary Evidence suggest possible reduced efficacy of some therapies esp. immunotherapy with reduced gut microbiome diversity and correlation with some antibiotic therapy. (Q12) P19<br>• Lack of mature data. (Q13) P2<br>• …need more data. (Q13) P30<br>• no standardization at the moment of the FMT, we do not know what we give an how it works, too much a black box intervention yet. (Q13) P27<br>• We need more data. (Q15) P2<br>• Lack of literature that they contribute to prevention or early recovery of neutropenia. (Q15) P16<br>• I have no enough data. (Q15) P24<br>• …my experience in this field and results of clinical studies. (Q15) P27<br>• …a patient with severe and prolonged neutropenia developed a fungemia that was related to probiotics that were being taken to regulate gut function. (Q15) P28<br>• …prior teaching but recognize limited clinical data to support this belief. (Q15) P32<br>• I have had patients who insist on taking probiotics develop bacteremia from the same bug. (Q15) P7<br>• *Depends on the threshold and setting, e.g. mildly neutropenic patient in outpatients would be fine with probiotics but I would not administer these in profoundly neutropenic patients in ICU, given the literature. (Q15) P10<br>• …prior probiotic study and one patient had sepsis. (Q15) P30<br>• Concern is gut barrier integrity. Need to confirm this prior to delivering a probiotic as the primary innate immune response (neutrophils) is significantly reduced. (Q15) P26<br>• *I do not have concerns from a safety perspective, but rather a scientific standpoint. We know that probiotics are limited in their diversity and load, and can in fact lead to the domination of the gut with a limited number of microbial species. As such, I would advocate for microbial interventions to be based on more complex microbial mixtures, e.g. FMT. (Q15) P12
	Significant-Cancer		• Evidence that the gut microbiome plays a role in getting cancer and response to treatment. (Q11) P21<br>• Especially for colorectal cancer (Q11) P36

**Significance of the microbiome**			• It's been proven that microbiome could be involved, not only in developing tumors, but also, in modulating the oncological response, toxicity, and other parameters related to our drugs (Q11) P17<br>• *I think huge impact on supportive care and late effects - inflammatory changes drive a lot of the complications (fatigue probably key) and we don't understand mechanism behind other factors like development of chronic pain (Q11) P35<br>• It would be interesting to see if there are carcinogen inactivating gut bacteria as opposed to carcinogen potentiating gut bacteria (Q11) P16
	Significant-Immune Response		• Immune mediation (Q11) P8<br>Ultimately, many disease states are influences by aberrant immune responses. The gut microbiome is the most influential factor shaping immune function, and as such it is highly likely that the microbiome indirectly impacts many disease states. (Q11) P8<br>• Inflammation plays a significant role in the development of cancer, and the gut microbiome exerts significant control over the immune system and inflammation. (Q11) P13<br>• immunity regulation. (Q11) P18<br>• Increasing evidence on role of gut in immune modulation. (Q11) P19<br>• Misbalance in the gut microbiome can increase patients' natural defense. (Q11) P25<br>• Gut microbiome interacts with mucosal innate immune mediators, leading to up or down regulation of immune and inflammatory responses with changes. There is now a lot of evidence to show gut microbiome is involved in not only cancer, but respiratory diseases, neurological conditions (coming about through gut-brain axis), diabetes, obesity, and a whole range of other diseases. It also plays a big role in treatments for diseases, affecting metabolism of many drugs (i.e. is required for activation of some drugs). (Q11) P26<br>• significant impact on immune regulation. (Q11) P32<br>• *I think huge impact on supportive care and late effects - inflammatory changes drive a lot of the complications (fatigue probably key) and we don't understand mechanism behind other factors like development of chronic pain. (Q11) P35<br>• a huge area to contact with environment stressors. (Q11) P31
	Significant-Both		• The microbiota is a key player in cancer pathogenesis, onset and progression. As regards breast cancer, its involvement includes the host's metabolism, immunity and hormonal balance through estrobolome (Q11) P3<br>• Its related to development of cancer but also with the imune capacity and the response to treatments. estrobolome (Q11) P23

			• It play(s) a role in immune modulation absorption of drugs in conjunction with bile acid can produce short or long chain fatty acids has both prevention and provocative role in colon cancer complex pathway, with intricacies still need to be understood (Q11) P34<br>• Indeed, scientifically nutrition management is important aspect in cancer patients, it also improves immunity of the patients. It improves the health of the patient. It has also very important role in palliative management (Q11) P22<br>• Potential pathogenesis connections, e.g. ETBF or F.nucleatum and CRC. Relationship via gut-immune axis and systemic inflammation, as well as metabolic regulation. Potential contribution to increased enterohepatic circulation re: oestrogen-related cancers via beta-glucuronidase. Contribution of gut microbiome to ICI response. Some connections more established than others. (Q11) P10<br>• It is everything. it likely determines health, wellness, disease, changes in inflammatory patterns, risks of disease and also reflects a disease modifiable factor. More should care about it! (Q11) P31
	Significant-Other Disease		• There is always lots of excitement around new fields, especially when the data are complex. I think the microbiome is important for what's happening in the GI tract but I am skeptical that the relationship between the microbiome and systemic disease is going to be meaningful. (Q11) P5<br>• *I'm not sure about cancer, but I do feel strongly about its' role in diseases like C diff. I have been concerned about the added antibiotics in the American food supply leading to increased rates of C diff in the general population. When I first started my career, we almost never saw C diff outside of a hospital setting. (Q11) P7
**Uncertainty**		• I really don't know. (Q11) P20<br>I don't see this relationship clinically. (Q11) p29<br>• Don't know they are strongly linked. (Q12) P11<br>• I really don't know. (Q12) P20<br>• If they work, we should use them. not my area of expertise. (Q13) P5<br>• …the issue is as a med oncologist, I don't decide on these issues. it becomes a matter for gastroenterology. it may be that this survey is targeting wrong physicians. (Q13) P18	• …antibiotics decisions are driven by pathogens and ID, not up to me. (Q12) P14<br>• *I'm not sure about cancer, but I do feel strongly about its' role in diseases like C diff. I have been concerned about the added antibiotics in the American food supply leading to increased rates of C diff in the general population. When I first started my career, we almost never saw C diff outside of a hospital setting. (Q11) P7<br>• Yes, but need more data first. (Q12) P15<br>• I don't know much about it. But It sounds risk in terms of cross infection or hipersensitivity reactions related to host defences. (Q13) P25
**Aspirations**		• I feel it will help to promote gut health and prevent GVHD. (Q13) P34<br>• I find it an interesting topic for research, with a great potential. (Q13) P17	• Maybe worth a shot. (Q12) P16<br>• The thought that probiotics are still opportunistic in nature. (Q15) P20

	• It is an interesting thought. (Q13) P20<br>• …we will be willing to do it if we get the opportunity for the same. (Q13) P22<br>• *All worth exploring - but within the context of a fully resourced clinical trial. (Q13) P35<br>• *Further clinical trials are required to identify the specific conditions required for each disease state, but this is a promising new technology for restoring microbiome following some sort of insult, ie antibiotics, disease, cancer treatment, etc. (Q13) P26<br>• I am not against this practice, but personally, I should study more about it. (Q15) P17<br>• I felt it will be feasible and at least non-harmful. (Q15) P34	
**Preferences**	• if a pt is myelo-suppressed and has sepsis, antimicrobial are life saving (Q12) P18<br>• Preference remains diet modification unless significant concerns re patients gut health where diet modification will be insufficient eg c.diff. (Q13) P19<br>• if done by expertised professionals. (Q13) P28	• Because antibiotics would alter the microbiome's ecosystem. (Q12) P24<br>• It depends on how neutropenic and what probiotics. (Q15) P8<br>• *Depends on the threshold and setting, e.g. mildly neutropenic patient in outpatients would be fine with probiotics but I would not administer these in profoundly neutropenic patients in ICU, given the literature. (Q15) P10<br>• Concern for infection. (Q15) P14
**Critiques**	• No concerns as long as it follows clearly established screening, administration and post-treatment monitoring pathways. (Q13) P10	• I do have a slight concern in regards to possible translocation of bacteria into the the bloodstream; however, it works wonders for the treatment of C diff (at least in the two patients we have treated). (Q13) P7<br>• Safety in immunocompromised patients. (Q13) P31<br>• concern for immunocompromised pts. (Q13) P32<br>• We over-prescribe antibiotics in NHS care routinely, e.g. yesterday someone suggested doxycycline for URTI in clinic because the patient had a sore throat. We need to be careful with antibiotic stewardship and consider risk-benefit properly. (Q12) P10<br>• We give antibiotics without considering the wider impact - and without then providing 'rescue' or even advice on restoring microbiome. (Q12) P35<br>• I think it is not sufficient as isolated matter. We need to improve de colon ambient. (Q13) P8<br>• Abhorrent, unless they are cultured in the lab and are free of faces! (Q13) P16<br>• *I do not have concerns from a safety perspective, but rather a scientific standpoint. We know that probiotics are limited in their

		diversity and load and can in fact lead to the domination of the gut with a limited number of microbial species. As such, I would advocate for microbial interventions to be based on more complex microbial mixtures, e.g. FMT. (Q15) P12<br>• Neutropenic pts should not be given bacterial loads. (Q15) P18

MICROBIOME: Current Practices and Perceptions

CONSENT TO TAKE PART IN A RESEARCH SURVEY
You are being invited to participate in a research study about perceptions and practices related to the gut microbiome in medical/pediatric oncology and hematopoietic stem cell transplant. This is a survey being conducted throughout MASCC (Multinational Association of Supportive Care in Cancer). We anticipate about 300 subjects will take part in the research.

The objective of this research project is to evaluate current practices related to the gut microbiome across centers and characterize perceptions of gut microbiome in research or clinical care in medical/pediatric oncology and stem cell transplant.

There are no known risks if you decide to participate in this research study, nor are there any costs for participating in the study. There are no direct benefits to you for taking part in this research, but you will be contributing to knowledge regarding the current practice variation, beliefs and gaps in knowledge related to the microbiome in patients with cancer. What we learn from this study will inform research priorities and clinical care, benefiting members and member organizations and people affected by cancer.

This survey is anonymous. If you choose to participate, no identifying information will be collected. The results of this study will be published in aggregated form in the future and presented at a MASCC annual meeting.

Your participation in this study is voluntary. If you choose to participate, please click "I agree" below. If you do not agree, please disregard this message and close out of the survey.

If you have any questions or concerns about completing the questionnaire or about being in this study, you may contact Dr. Freedman at +1-267-425-5822 or at freedmanj@chop.edu.

The Children's Hospital of Philadelphia Institutional Review Board has reviewed our request to conduct this project and deemed this exempt from full review.

1) By beginning this research, I acknowledge that I am 18 years of age or older and have read and understand the information. I agree to take part in the research. I understand that completion of the survey implies consent.

- ◯ I Agree
- ◯ I Do Not Agree

Current Practices

Please answer these questions to the best of your ability.

Demographic Information

In what country are you currently living/working?

____

What is your primary occupation?

- ◯ Physician
- ◯ Nurse
- ◯ Dentist/Oral Health Specialist
- ◯ Advanced Practice Provider
- ◯ Allied Health Professional (Nutritionist/Dietician, Occupational Therapist, Physiotherapist, etc.)
- ◯ Pharmacist
- ◯ Academic/Researcher
- ◯ Student

What is your primary work location?

- ◯ Hospital
- ◯ University or Research Institute
- ◯ University Hospital
- ◯ Private Clinic
- ◯ Community Based Practice
- ◯ Other

Other work location

____

If you practice clinically, what is your primary specialty?

- ◯ Adult Oncology
- ◯ Paediatric Oncology
- ◯ Adult Stem Cell Transplantation
- ◯ Paediatric Stem Cell Transplantation
- ◯ All of the above
- ◯ I do not practice clinically

Other primary work location:

____

Approximately how many new patients/diagnoses are there each year at your center?

____

Approximately how many stem cell transplants are performed each year at your center?

____

Does your centre have a specific immunocompromised ID service available for consults?

- ◯ Yes
- ◯ No

Does your centre have an antimicrobial stewardship program?

- ◯ Yes
- ◯ No

If you do research, what is/are your research interest(s)

____

Perceptions

Do you feel the gut microbiome plays a big role in disease states such as cancer?	◯ Yes<br>◯ No
Please explain your thoughts	__________________
Do you think the gut microbiome should be taken into consideration when making decisions around prophylaxis or empiric antibiotics?	◯ Yes<br>◯ No
Please explain your thoughts	__________________
Do you have any personal objections or concerns regarding faecal microbiota transplantation (FMT)?	◯ Yes<br>◯ No
Please explain your perspective(s)	__________________
Which method of FMT administration do you consider most preferable for your patients (for researchers - your patient population of interest)?	◯ Colonoscopically<br>◯ Rectal enema<br>◯ Nasogastric tube<br>◯ Capsules<br>◯ Other
Other (please describe):	__________________
Why do you prefer this method?	__________________
What practical aspects would be important considerations in adopting an FMT service in your center? For example, administration method, source of stool (autologous vs donor), timing of administration.	__________________
Do you have concerns about probiotics being delivered to neutropenic patients?	◯ Yes<br>◯ No
What has informed this perspective?	__________________
Are there any other important factors that you believe contraindicate microbial-based interventions?	__________________
Do you have concerns about FMT being delivered to neutropenic patients?	◯ Yes<br>◯ No
